# A model for prediction of a twin live birth following *in vitro* fertilization–embryo transfer in women of advanced age

**DOI:** 10.3389/fmed.2026.1789377

**Published:** 2026-10-13

**Authors:** Siyue Xu, Ruolei Wang, Cuilian Zhang, Meng Li, Shaodi Zhang

**Affiliations:** 1 Reproductive Medicine Center, Henan Provincial People’s Hospital, Zhengzhou, China; 2 State Key Laboratory of Reproductive Medicine and Offspring Health, Nanjing Medical University, Nanjing, China

**Keywords:** advanced age, IVF, model, transferred embryo number, twin live birth

## Abstract

**Introduction:**

The aim of this study was to construct a model for prediction of the probability of a twin live birth in women of advanced age *in vitro* fertilization and embryo transfer cycles.

**Methods:**

This retrospective analysis included 10,089 transfer cycles from women aged ≥35 years, stratified by whether they had a live twin birth. Taking a twin live birth as the positive outcome (Y = 1) and cycles with a singleton or no live birth as the negative outcome (Y = 0). A multivariate logistic regression analyses was built based on candidate variables, followed by nomogram construction. The discrimination ability of the model was assessed by the area under the receiver-operating characteristic curve, with 500 bootstrap resamples for internal validation.

**Results:**

There were significant differences in age, endometrial thickness, number of usable embryos, number of embryos transferred, and embryo type between the groups (all *P* < 0.05). Multivariate logistic regression identified the following five predictors of a twin live birth: age (adjusted odds ratio [aOR] 0.69, 95% confidence interval [CI] 0.66–0.73, *P* < 0.001), endometrial thickness ≥7 mm (aOR 2.79, 95% CI 1.30–5.99, *P* = 0.009), blastocyst transfer (aOR 1.88, 95% CI 1.36–2.60, *P* < 0.001), two good quality embryos (aOR 2.20, 95% CI 1.71–2.82, *P* < 0.001), and two embryos transferred (aOR 19.45, 95% CI 11.57–32.69, *P* < 0.001). The nomogram built on these variables yielded an area under the curve of 0.8462. Bootstrap internal validation (500 resamples) produced a corrected area under the curve of 0.8443.

**Conclusions:**

The likelihood of a twin live birth correlated positively with two embryos transferred, two good quality embryos, blastocyst transfer, and endometrial thickness ≥7 mm, and correlated negatively with maternal age.

## Introduction

Multiple pregnancies is a common complication of assisted reproductive technology (ART). Embryo implantation and clinical pregnancy rates have increased significantly with the development of clinical and laboratory techniques for ART. In most reproductive medicine centers in China, there is still a relatively high proportion of multiple embryo transfers (2–3 embryos), resulting in an increased multiple pregnancy rate (1, 2). Data from the Society for Assisted Reproductive Technology show that 36.2% of pregnant women aged 38–40 years who underwent multiple embryo transfer in frozen–thawed cycles in 2023 had multiple pregnancies, whereas the multiple pregnancy rate after single embryo transfer (SET) in the same age group was only 1.1% (3). Various pregnancy related complications, including preterm birth and low birth weight associated with multiple pregnancies, can threaten the life and health of both mother and infants (4). Therefore, there is an urgent need to reduce the multiple pregnancy rate. With implementation of SET, both twin and multiple pregnancy rates have declined (5).

Studies have shown that reducing the number of embryos transferred helps to reduce the risk of multiple pregnancies in younger women (6, 7). However, no consensus has been reached on whether SET should be used in women of advanced age. The European Society of Human Reproduction and Embryology recommends that women undergoing ART (including women of advanced age) should receive elective SET (8). Nevertheless, there are some challenges when performing SET in women of advanced age. First, clinical pregnancy and live birth rates are low after SET according to data from the Society for Assisted Reproductive Technology in the USA for 2023, with the live birth rate being 11.6% for women aged >38 years, 3.2% for those aged ≥41 years, and only 0.8% for those aged >42 years (3). Second, women of advanced age with poor clinical outcomes after SET often require multiple embryo transfers to achieve a live birth, and repeated ART procedures increase the financial burden and time costs for patients. Therefore, there continues to be resistance to implementing SET in older patients. The risk of perinatal complications, including obstetric and neonatal problems, is greatly increased in older patients with multiple pregnancy. Therefore, it is particularly important to reduce the likelihood of multiple deliveries in older patients who have undergone ART (9). Several studies have investigated the impact of embryo transfer strategies on clinical outcomes in older patients. For example, data from 1,284 frozen blastocyst transfer cycles in women aged ≥35 years showed that live birth rates were comparable between double blastocyst and single blastocyst transfer in women aged 35–37 years, whereas the live birth rate was higher after double blastocyst transfer than after single blastocyst transfer in women aged 38–42 years (10). Another study found no significant difference in the pregnancy rate or multiple pregnancy rate but observed a significantly increased live birth rate in women aged ≥35 years when two poor quality blastocysts were transferred rather than a single poor quality blastocyst (11). The above mentioned studies focused only on the effects of various numbers of blastocysts or their quality on clinical outcomes in older patients. Although it is well known that age, endometrial thickness, type of embryo, and embryo quality are key indicators affecting clinical outcomes, none of the previous studies have included in depth analyses.

In this study, we used candidate factors to construct a prediction model for twin live birth in women of advanced age, and analyzed multiple indicators of ART assisted pregnancy in women aged ≥35 years to guide individualized embryo transfer strategies for elderly patients.

## 2 Materials and methods

### 2.1 Study population

The study included 12,546 transfer cycles from women of advanced age who underwent assisted reproductive treatment with *in vitro* fertilization or intracytoplasmic sperm injection–embryo transfer (IVF/ICSI-ET) at the Reproductive Center of Henan Provincial People's Hospital between January 2016 and May 2023. Women were included if they were aged ≥35 years and undergoing fresh-cycle IVF/ICSI-ET or frozen–thawed embryo transfer. The following exclusion criteria were applied: chromosomal abnormalities in either partner and preimplantation genetic testing transfer cycles (*n* = 491); oocyte donation transfer cycles (*n* = 25); transfer cycles lost to follow-up (*n* = 60); sequential transfer cycles (*n* = 373); transfer cycles with missing key predictor variable data (*n* = 1,215); transfer cycles with scar pregnancy (*n* = 1), ectopic pregnancy (*n* = 54), or concurrent intrauterine and ectopic pregnancy (*n* = 8); and transfer cycles with pregnancies with a reduced fetal number, including ongoing pregnancies with a history of fetal reduction or spontaneous embryonic reduction (*n* = 230). Pregnancies in older women carry a risk of spontaneous fetal loss, and both spontaneous and selective fetal reduction can result in a mismatch between the number of fetuses *in utero* and the delivery outcome. These cases were excluded to allow accurate analysis of the direct impact of number of transferred embryos on the number of live births. A sensitivity analysis was performed for confirmation of robustness; the full data are shown in Supplementary Table S1 and Figures S1, S2, S3a, and S3b. Finally, the study included 10,089 transfer cycles from 6,115 women. These cycles comprised 387 twin live births and 9,702 cycles without twin live births (7,499 with no live-birth events and 2,203 singleton live births).

The study was approved by the Reproductive Medicine Ethics Committee of Henan Provincial People's Hospital (SYSZ-LL-2021091501). The ethics committee authorized access to de-identified clinical data and waived the need for written informed consent. The flow of patients through the study is shown in Figure 1.

**Figure 1:**
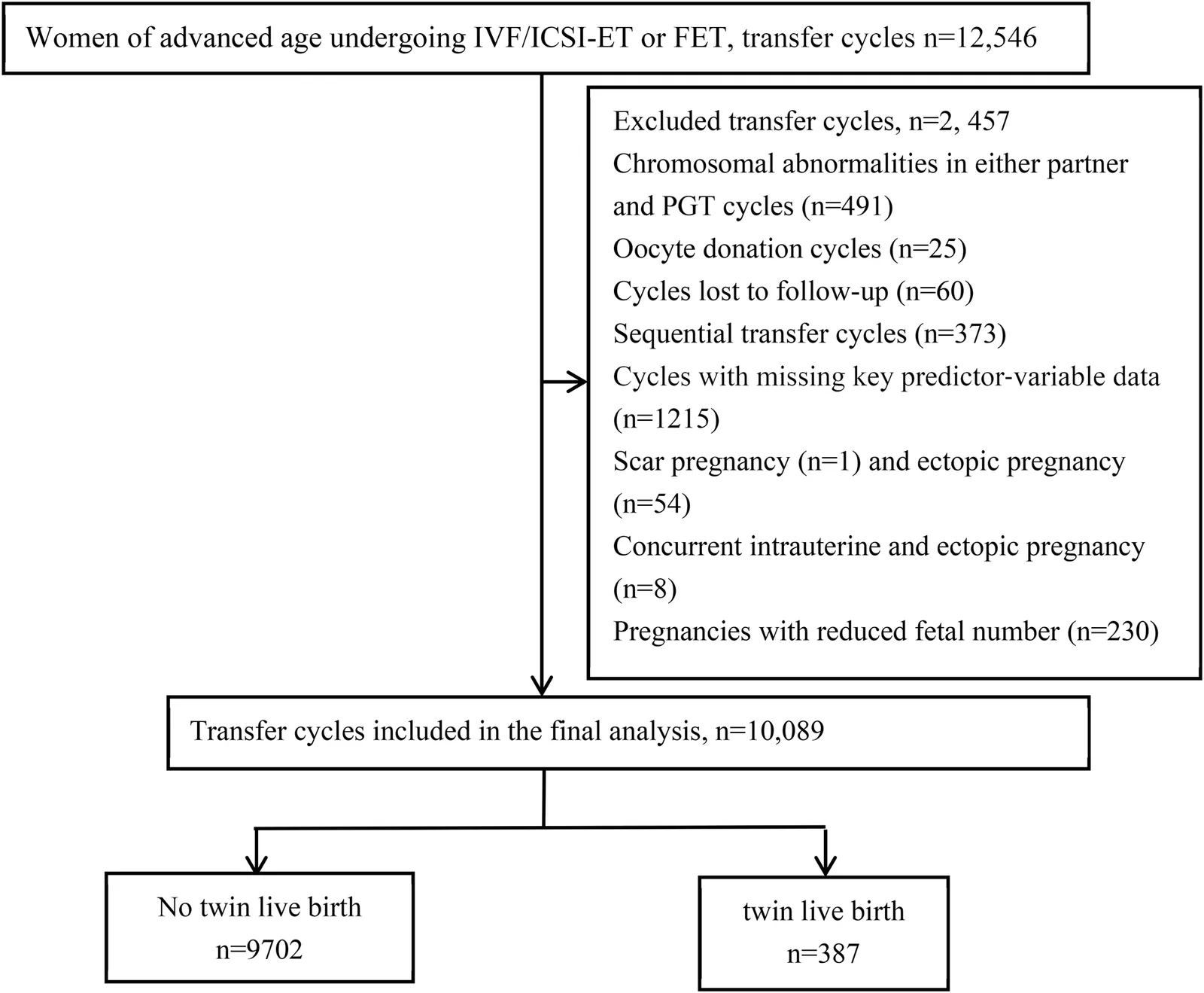
Flowchart showing the flow of patients through the study. FET, frozen embryo transfer; IVF, *in vitro* fertilization; ICSI-ET, intracytoplasmic sperm injection–embryo transfer; PGT, preimplantation genetic testing.

### 2.2 Protocols

#### 2.2.1 Controlled ovarian stimulation

##### 2.2.1.1 GnRH agonist protocol

Luteal phase short-acting gonadotropin-releasing hormone (GnRH) agonist long protocol.

In this study, patients with polycystic ovary syndrome had menstrual irregularities, oligo-ovulation, or anovulation. Tablets containing 2 mg cyproterone acetate + 35 µg ethinyl estradiol (Diane-35; Bayer AG, Leverkusen, Germany) were taken daily from the start of the menstrual period, with daily administration of a GnRH agonist commencing when 4–6 tablets remained. Dose adjustment involved subcutaneous injection of a short-acting GnRH agonist, namely, triptorelin acetate (Decapeptyl; French Ferring Pharmaceuticals, Gentilly, France; 0.1 mg/per injection). After administration of Decapeptyl 0.1 mg/day for 14–18 days, hormone levels and ultrasound scans were checked. When the downregulation criteria were met, gonadotrophin stimulation and a GnRH agonist (0.05 mg/day) were initiated. The criteria for downregulation were a serum follicle-stimulating hormone level <5 mIU/mL, a luteinizing hormone level <5 mIU/mL, an estradiol level <50 pg/mL, a progesterone level <1 ng/mL, endometrial thickness confirmed by ultrasonography to be <5 mm, a follicle diameter of <10 mm in both ovaries, and no luteal cysts. The follicular phase long-acting protocol followed the method previously used at our center (12).

The starting dose of gonadotrophin was determined based on the patient's age, body mass index, serum anti-Müllerian hormone level, antral follicle count, and previous ovarian response. Adjustments to the gonadotrophin dose were made during controlled ovarian stimulation according to follicle size and number and hormone levels. When follicles with a diameter of ≥18 mm accounted for 40%–60% of the dominant follicles (with a diameter of ≥14 mm), ovulation was triggered with 250 μg of Ovidrel (Merck, Rahway, NJ, USA) or a 6,000–10,000 IU intramuscular injection of human chorionic gonadotropin (hCG; Zhuhai Lizhu Pharmaceuticals, Zhuhai, China). Follicular aspiration was performed 36–38 h after the ovulation trigger.

##### 2.2.1.2 GnRH antagonist protocol

Gonadotropin was injected from the second or third day of menstruation, and the starting dose was the same as above. Follicular size and hormone levels were monitored after 4 or 5 days of treatment with gonadotrophin. A GnRH antagonist was initiated at a daily dose of 0.25 mg with detection of at least one 14–15 mm follicle or a dominant follicle diameter of 12 mm and a serum estradiol concentration of >300 pg/mL. The same dose was administered until the day of administration of hCG. When at least two follicles measured ≥18 mm or three follicles measured ≥17 mm, hCG was administered subcutaneously at a dose of 4,000–10,000 IU. Triggering with 0.1–0.2 mg of short-acting Decapeptyl and administration of hCG 1,000–2,000 IU could be used if there was a high risk of ovarian hyperstimulation syndrome. Oocyte retrieval guided by vaginal ultrasound was performed 35–36 h after administration of hCG.

#### 2.2.2 Embryo transfer and luteal phase support

The criteria for fresh embryo transfer included an endometrial thickness of ≥8 mm with uniform echo, serum progesterone <1.5 μg/L, absence of infection, a tendency towards ovarian hyperstimulation syndrome, or any specific medical history. One or two embryos were transferred at the cleavage stage. One single embryo was chosen for transfer at the blastocyst stage. The remaining embryos or blastocysts that met the cryopreservation criteria were vitrified for future frozen–thawed embryo transfer. Women undergoing fresh transfer were treated with oral dydrogesterone (Duphaston; Abbott Laboratories, Abbott Park, IL, USA) at a dose of 10 mg twice daily in conjunction with a slow-release vaginal progesterone gel (Crinone, Merck Serono, Darmstadt, Germany) at a dose of 90 mg once daily, starting on the day of oocyte retrieval.

Frozen–thawed embryo transfer was performed in patients who did not achieve a live birth after a fresh transfer and had remaining embryos or did not undergo a fresh cycle transfer. The decision to use a hormone replacement therapy (HRT) protocol and an ovulation induction protocol was based on the patient's menstrual and ovulation status.

##### 2.2.2.1 HRT protocol

This was initiated with oral estradiol valerate at 4–8 mg day (Progynova; Bayer AG) on days 2–4 of the menstrual cycle to prepare the endometrium. If the endometrium was found to be thin, further treatment included vaginal administration of Femoston (Abbott Laboratories; 2 mg/tablet) at 2 mg once or twice per day. When estrogen had been used for 11–20 days and the endometrial thickness had reached ≥8 mm or matched the thickness on the oocyte retrieval trigger day of the cycle, Duphaston (10 mg twice a day) and vaginal progesterone gel (Crinone, 90 mg once daily) were added. Day 3 cleavage-stage embryos were transferred on day 4, and blastocysts from day 5–6 were transferred on day 6 after initiation of progesterone.

##### 2.2.2.2 Downregulation of the HRT protocol

On days 2–4 of the menstrual cycle, a long-acting GnRH agonist (the same as the one administered in the early follicular-phase long-acting long protocol) was administered at a dose of 3.75 mg. After 14–21 days, transvaginal ultrasound and sex hormone levels were reassessed. Estrogen was initiated when the criteria for adequate downregulation were met. The medications and duration of use, endometrial criteria, and luteal support were the same as those in the HRT cycle.

##### 2.2.2.3 Natural cycle

For patients with regular menstrual cycles, an ultrasound examination was performed on day 11–12 to monitor endometrial thickness and follicle size. When the follicle reached ≥16 mm, follicles were monitored daily until ovulation. If ovulation had not occurred when the follicle was ≥18–20 mm, hormone levels were monitored; hCG 5,000–10,000 IU was injected to induce ovulation if the serum progesterone level was <1 ng/mL. Cleavage-stage embryos were transferred on day 3 after ovulation, and blastocysts were transferred on day 6. Luteal support was provided after ovulation.

### 2.3 Assessment of embryos

Each cleavage-stage embryo was graded according to its speed of development, degree of fragmentation, and evenness of blastomere size. Day 3 embryos with consistent or slightly uneven blastomere sizes, regular or irregular shapes, and fragmentation ≤25% were defined as usable for transfer. Blastocysts were graded according to the Gardner scoring system (13).

A good quality embryo were defined as follows: day 3 cleavage-stage with 7–9 cells, fragmentation ≤20%, and equal blastomere size (14); and day 5 or 6 blastocysts with an expansion grade ≥3 and graded as AA, AB, BA, or BB. Poor quality embryos were defined as follows: day 3 cleavage-stage with <7 or >9 cells and fragmentation >20%; and day 5 or 6 blastocysts with an expansion grade ≥3 and graded as BC or CB, AC, or CA.

### 2.4 Follow-up and outcome measures

Serum hCG levels were measured 14 days after embryo transfer. Patients with a positive hCG result underwent transvaginal ultrasound examination 28–35 days after transfer. Clinical pregnancy was defined as visualization of a gestational sac (intrauterine or extrauterine) on ultrasound. The presence of two or more fetuses in the uterine cavity was defined as multiple pregnancy. The presence of one fetus was defined as singleton pregnancy.

A singleton live birth refers to delivery of one live fetus at ≥22 weeks' gestation, and a twin live birth refers to delivery of two live fetuses from ≥22 weeks' gestation onwards.

### 2.5 Statistical analysis

All statistical analyses were performed using Empower Stats built on R software and Posit Cloud (R language). Normally distributed continuous variables are shown as the mean ± standard deviation, skewed data as the median [interquartile range], and categorical data as the number (percentage). Two group comparisons were performed using the independent *t*-test, Mann–Whitney *U* test, or chi-squared test as appropriate.

After screening, the rate of missing data for each covariate was below 5%. Scattered minor missing values were imputed via the median for continuous variables and the mode for binary variables. Given the low proportion of missing data (<5%) and the assumption of a MCAR (missing completely at random) mechanism, single imputation was used to reduce analytical bias. The variance inflation factor was <3 for all variables, ruling out obvious multicollinearity in the multivariate model. Additional sensitivity analysis was conducted using multiple imputation. Comparisons of multivariable regression estimates from multiply-imputed datasets and the primary dataset indicated that our main findings were robust (Supplementary Table 8).

All candidate predictors were pre-specified before statistical analysis, based on previously published ART studies on twin live birth risk and clinical biological plausibility. Univariate logistic regression analyses were performed only for descriptive exploratory purposes, and were not used as a criterion for variable inclusion. The binary outcome was defined as a twin live birth (Y = 1) versus a non-twin live birth (Y = 0). Subjects with 0 or 1 high quality embryos were merged into one reference group owing to the equivalent risks of a twin live birth (Supplementary Table S2).

The number of transferred embryos was regarded as an adjustable treatment variable rather than a fixed baseline factor. The present model calculates personalized pre-transfer probabilities of a twin live birth under SET or double embryo transfer (DET) to optimize embryo transfer regimens. An interaction term of embryo type (cleavage-stage versus blastocyst) × number of transferred embryos was included in the multivariate regression model to test for effect modification. No statistically significant interaction was observed; accordingly, this interaction term was excluded from the final main model used for nomogram construction. We also performed two subgroup regression stratifications (SET/DET and cleavage/blastocyst) for confirmation of robustness; relevant interaction and stratified outcomes are listed in Supplementary Tables S2–S5.

Candidate factors were used to construct a prediction model for twin live birth. The discriminative performance of the model was evaluated using the area under the receiver-operating-characteristic curve (AUC). Bootstrap internal validation with 500 resamples was implemented in R and Empower Stats. The optimism-corrected AUC, calibration curve, and Brier score were derived from out-of-bag samples. All statistical tests were two-tailed, and a *P* value < 0.05 was regarded as statistically significant.

## 3 Results

### 3.1 General characteristics of the study groups

Table 1 presents the baseline characteristics, clinical ovarian stimulation and laboratory related indicators, and outcome measures for the two groups. Significant between group differences were observed in age, basal follicle stimulating hormone, anti-Müllerian hormone, endometrial thickness, number of oocytes retrieved, number of usable embryos, type of infertility, number of good quality embryos, number of transferred embryos, and embryo type (*P* < 0.05). There was no statistically significant between group difference in body mass index, duration of infertility, basal estradiol level, or basal luteinizing hormone level.

**Table 1:** Comparison of baseline characteristics according to live birth outcome.

Live birth outcome	No twin live birth	Twin live birth	*P*-value
*N*	9,702	387	
Age	38.81 ± 3.17	36.62 ± 1.81	<0.001
BMI	23.68 ± 3.11	23.52 ± 2.95	0.548
Duration of infertility	3.00 (1.50–6.00)	3.00 (1.50–6.50)	0.471
Basal FSH (mIU/mL)	7.99 ± 3.29	7.24 ± 2.26	<0.001
Basal LH (mIU/mL)	5.08 ± 2.38	5.19 ± 2.50	0.593
Basal E2 (pg/mL)	40.29 ± 18.53	37.80 ± 16.06	0.053
AMH (ng/mL)	1.78 (0.88–3.04)	2.48 (1.48–4.00)	<0.001
Number of oocytes retrieved	6.00 (4.00–11.00)	8.00 (6.00–12.00)	<0.001
Number of usable embryos			<0.001
≤4	7,079 (72.96%)	248 (64.08%)	
>4, ≤9	2,214 (22.82%)	108 (27.91%)	
>9, ≤15	365 (3.76%)	28 (7.24%)	
>15	44 (0.45%)	3 (0.78%)	
Endometrial thickness(mm)			<0.001
<7	588 (6.06%)	7 (1.81%)	
≥7	9,114 (93.94%)	380 (98.19%)	
Type of infertility			<0.001
Secondary infertility	7,960 (82.04%)	286 (73.90%)	
Primary infertility	1,742 (17.96%)	101 (26.10%)	
Number of good quality embryos transferred			<0.001
≤1	7,014 (72.29%)	160 (41.34%)	
2	2,688 (27.71%)	227 (58.66%)	
Transferred embryo number			<0.001
1	4,119 (42.46%)	17 (4.39%)	
2	5,583 (57.54%)	370 (95.61%)	
Embryo type			<0.001
Cleavage-stage embryo	6,569 (67.71%)	292 (75.45%)	
Blastocyst	3,133 (32.29%)	95 (24.55%)	

AMH, anti-Müllerian hormone; BMI, body mass index; E2, estradiol; FSH, follicle-stimulating hormone; LH, luteinizing hormone.

### 3.2 Univariate and multivariate regression analyses of factors associated with a twin live birth

The results of the univariate and multivariate analyses of all candidate factors are provided in Table 2. After adjustment for confounders, older age(adjusted OR [aOR] 0.69, 95% confidence interval [CI] 0.66–0.73, *P* < 0.001) was significantly associated with a lower probability of twin live birth, whereas endometrial thickness ≥7 mm (aOR 2.79, 95% CI 1.30–5.99, *P* = 0.009), blastocyst transfer (aOR 1.88, 95% CI 1.36–2.60, *P* < 0.001), two good quality embryos (aOR 2.20, 95% CI 1.71–2.82, *P* < 0.001), and two embryos transferred (aOR 19.45, 95% CI 11.57–32.69, *P* < 0.001) significantly increased the likelihood of a twin live birth.

**Table 2:** Univariate and multivariate analyses of factors associated with a twin live birth.

Variable	Univariate analysis		Multivariate analysis	
	OR(95%CI)	*P*-value	aOR(95%CI)	*P*-value
Age	0.71 (0.68, 0.75)	<0.001	0.69 (0.66, 0.73)	<0.001
Type of infertility				
Secondary infertility	1		1	
Primary infertility	1.61 (1.28, 2.04)	<0.001	1.03 (0.80, 1.32)	0.832
Basal FSH (mIU/mL)	0.91 (0.88, 0.95)	<0.001	0.97 (0.92, 1.01)	0.127
Basal E2 (pg/mL)	0.99 (0.99, 1.00)	0.009	0.99 (0.99, 1.00)	0.105
AMH (ng/mL)	1.12 (1.08, 1.16)	<0.001	1.04 (0.99, 1.09)	0.119
Number of oocytes retrieved	1.05 (1.03, 1.07)	<0.001	0.99 (0.96, 1.02)	0.632
Number of usable embryos				
≤4	1		1	
>4, ≤9	1.39 (1.11, 1.75)	0.005	1.00 (0.75, 1.34)	0.990
>9, ≤15	2.19 (1.46, 3.28)	<0.001	1.74 (0.98, 3.08)	0.058
>15	1.95 (0.60, 6.31)	0.267	2.77 (0.71, 10.80)	0.143
Endometrial thickness(mm)				
<7	1		1	
≥7	3.50 (1.65, 7.43)	0.001	2.79 (1.30, 5.99)	0.009
Number of good quality embryos transferred				
1	1		1	
2	3.70 (3.01, 4.55)	<0.001	2.20 (1.71, 2.82)	<0.001
Transferred embryo number				
1	1		1	
2	16.06 (9.86, 26.15)	<0.001	19.45 (11.57, 32.69)	<0.001
Embryo type				
Cleavage-stage embryo	1		1	
Blastocyst	0.68 (0.54, 0.86)	0.001	1.88 (1.36, 2.60)	<0.001

The odds ratio for each continuous variable corresponds to a 1-unit increment in its value. Reference groups: transferable embryos ≤4, secondary infertility, endometrial thickness <7 mm; group 1 was used as the reference for other binary variables. AMH, anti-Müllerian hormone; E2, estradiol; FSH, follicle-stimulating hormone.

### 3.3 Model for prediction of a twin live birth

Logistic regression analysis identified the predictors of a twin live birth to include age, number of embryos transferred, type of embryos transferred, endometrial thickness, and number of good quality embryos. These independent variables were incorporated into a nomogram. The details are shown in Figure 2. The performance of the model was evaluated by calculating the AUC. The twin live birth risk prediction model was expressed using the logit equation [logit(*p*) = ln(*p*/1-*p*) = 8.51323–0.37756 × age* +* 2.95569 × (transferred embryo number = 2) – 0.76533 × (cleavage-stage embryo) + 1.08475 × (endometrial thickness ≥7 mm) – 0.82053 × (number of good quality embryos = 0/1). In the formula, *p* denotes the predicted probability of a twin live birth; the AUC was 0.8462, with a sensitivity of 0.8738 and a specificity of 0.7137 from the original apparent dataset, indicating that the model had good predictive ability.

**Figure 2:**
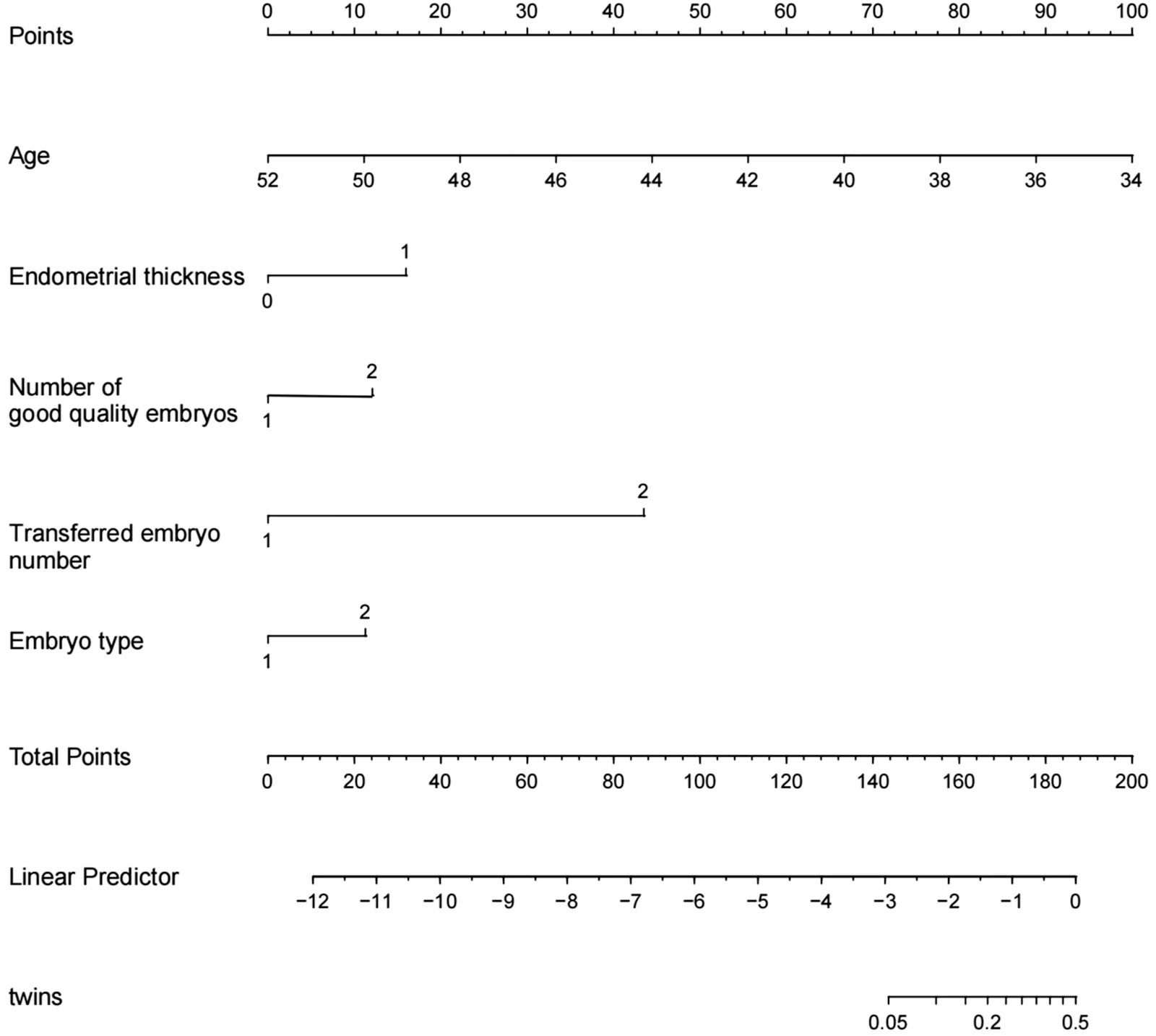
Nomogram for prediction of the likelihood of a twin live birth. Endometrial thickness: 0 to <7 mm; 1 to ≥7 mm. Number of good-quality embryos: 1, 0/1 good-quality embryos; 0, 2 good-quality embryos. Embryo type: 1, cleavage-stage embryo; 0, blastocyst. Transferred embryo number: 0, 1 embryo; 1, 2 embryos.

The nomogram was internally validated using 500 bootstrap resamples. Details are shown in Figure 3. The results are summarized below. In terms of discrimination, the apparent AUC of the model was 0.8462 with an optimism value of 0.0019; the optimism-corrected C-index (mathematically equivalent to the AUC for binary outcomes) was 0.8443 (95% CI: 0.8438–0.8460). At the optimal threshold identified by maximum Youden's index, the sensitivity, specificity, positive predictive value, and negative predictive value were 0.8520, 0.7198, 0.1072 and 0.9919, respectively. In terms of calibration performance averaged across 500 bootstrap replicates, the mean calibration intercept was −0.0290 and the mean calibration slope was 0.9888. The apparent Brier score quantifying the overall predictive error was 0.0340 with negligible optimism (−0.0002, slight sampling-driven negative bias); the optimism corrected Brier score was 0.0342 (95% CI: 0.0339–0.0343). The optimism-corrected calibration curve is shown in Figure 4a. Within the clinically relevant low-to-intermediate risk range (predicted probability: 0–0.2), the fitted curve was in good agreement with the ideal diagonal line, with only mild overestimation of twin live birth risk at predicted probabilities above 0.2. The minor deviation occurred in this high-probability region, where relatively few study cycles were present, as shown by the rug plot at the bottom of the figure. When stratified by risk subgroups, predicted probabilities closely agreed with observed twin live birth rates across the low-risk (0–0.1) and intermediate-risk (0.1–0.2) intervals, which cover the vast majority of patients in our cohort given the overall baseline twin live birth rate of 3.8%. The slight deviation observed at predicted probabilities greater than 0.2 occurred in a relatively small subset of high-risk cycles and had limited influence on the overall clinical utility of the model. Decision curve analysis was performed across thresholds of 0%–100% (Figure 4b). Over the threshold probability range of 0%–20%, the model provided higher net benefit relative to both the treat-all and treat-none strategies.

**Figure 3:**
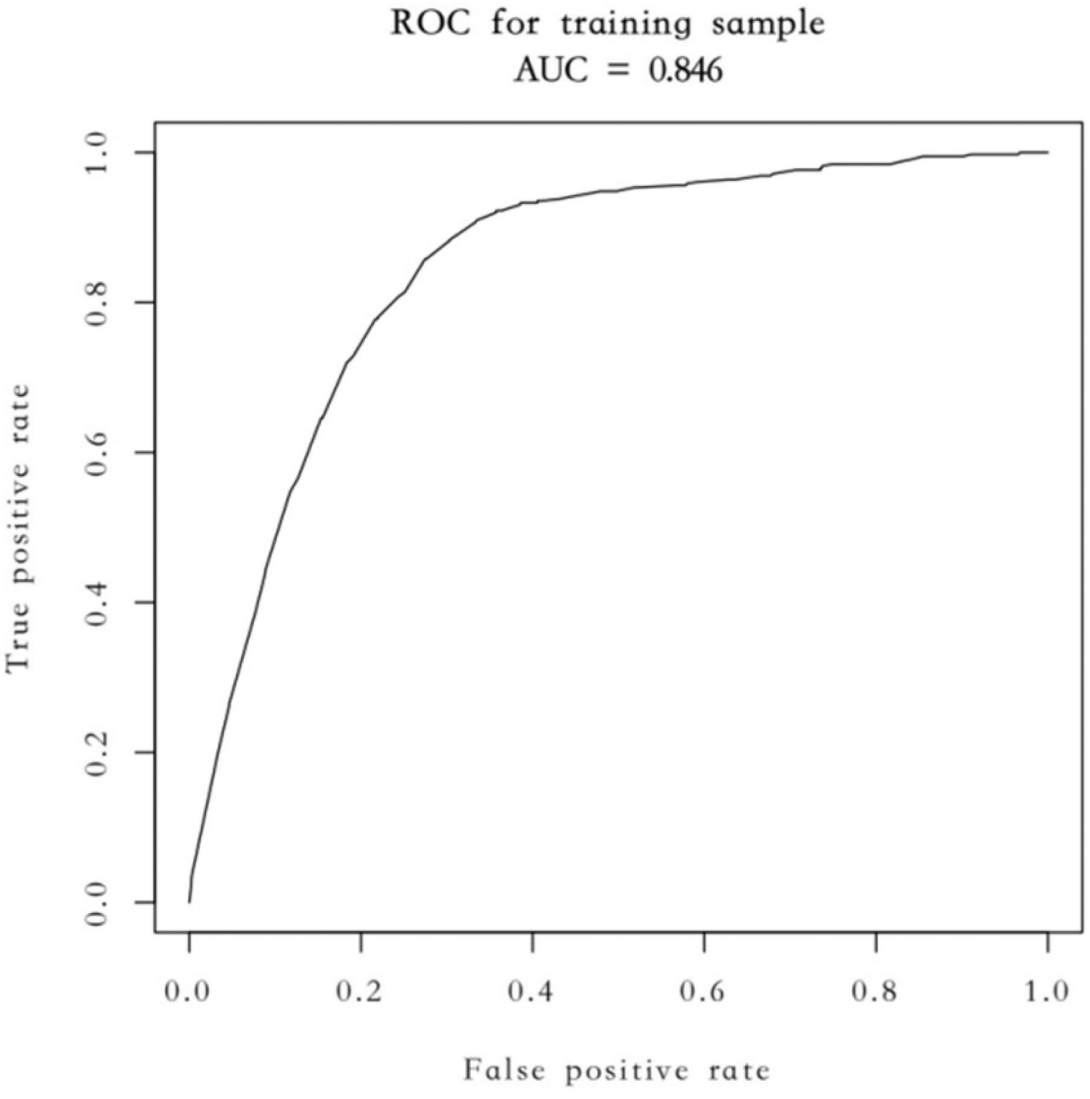
ROC curve for assessment of the accuracy of prediction of a twin live birth. AUC, area under the curve; ROC, receiver-operating characteristic.

**Figure 4:**
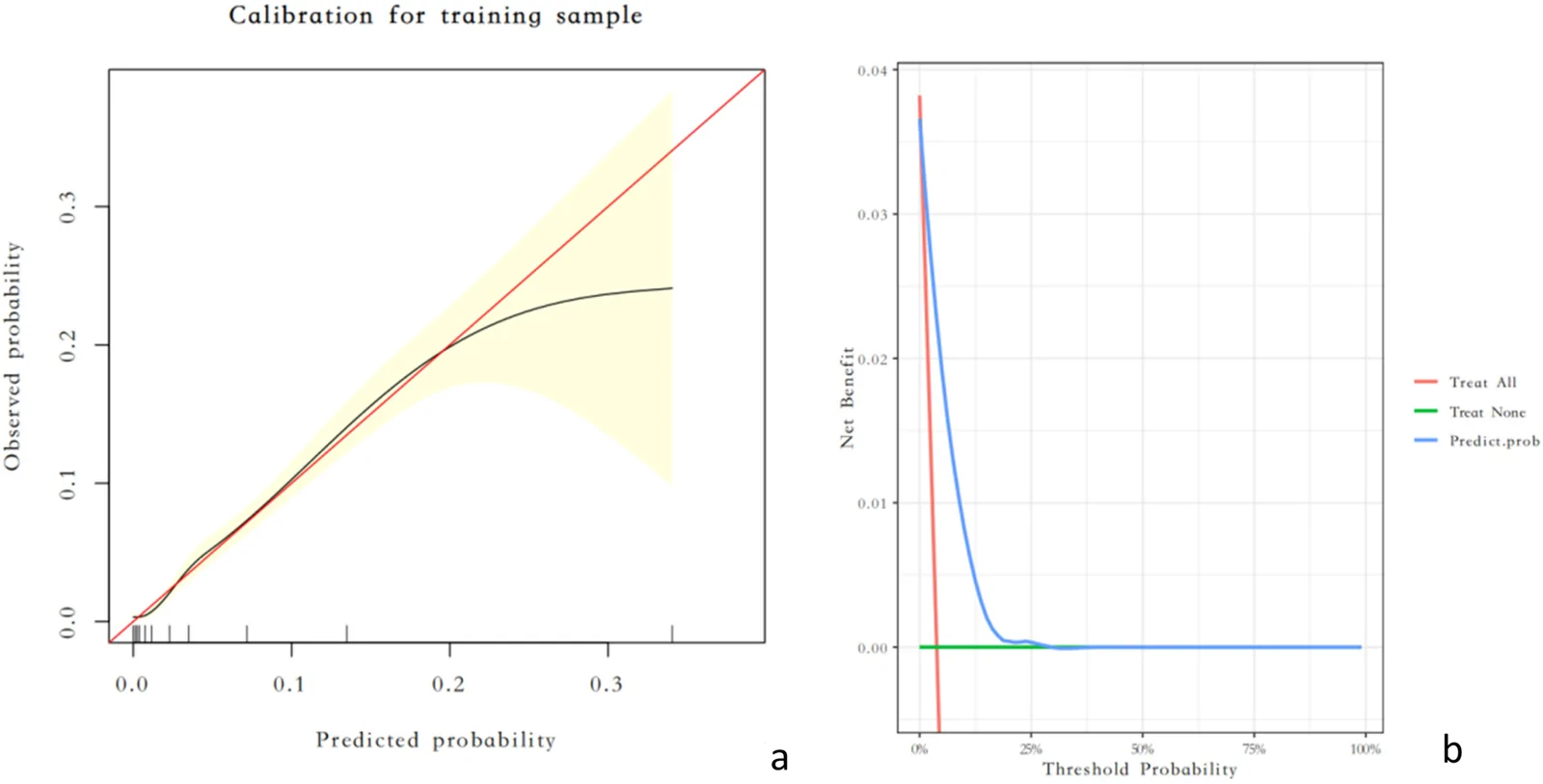
Calibration curve and decision curve analysis (DCA) of the predictive model in the training cohort. **(a)** Calibration curve for the training sample. This curve was used to assess model calibration. The probability of a twin live birth predicted by the model is shown on the *x*-axis. The observed probability of a twin live birth is shown on the *y*-axis. The red diagonal line represents the ideal calibration, with the black fitted curve enclosed by yellow shading representing the 95% confidence interval. **(b)** DCA for the training cohort. The blue curve indicates the net benefit of the predictive model. The red curve indicates universal embryo transfer. The green curve indicates no embryo transfer.

## 4 Discussion

In this large sample retrospective cohort study of women aged ≥35 years, we identified predictive factors correlated with the likelihood of a twin live birth through univariate and multivariate analyses and developed a prediction model incorporating age, number and type of embryos transferred, endometrial thickness, and number of good quality embryos. This model was used to predict a twin live birth in older patients before embryo transfer and to guide clinicians in selecting appropriate transfer strategies to reduce the likelihood of a twin live birth. To our knowledge, this is the first prediction model for a twin live birth to be developed for women aged ≥35 years. We found that DET is a strong risk factor for a twin live birth, which is consistent with a previous report (15). We also found that increased endometrial thickness, a higher number of good quality embryos, and blastocyst transfer increased the risk of a twin live birth, whereas increasing age reduced this risk.

In this study, DET was applied in 74.6% (5,121/6,861) of cleavage-stage cycles and SET in 74.2% (2,396/3,228) of blastocyst cycles. Uneven distribution of the transferred embryo count caused confounding at baseline and reversed univariate and multivariate associations. Without adjusting for this clinical decision variable, blastocyst cycles showed a lower crude risk of a twin live birth as a result of the dominance of SET. After adjustment, the superior implantation potential of blastocysts became evident, making blastocyst transfer an independent risk factor in multivariate models. The non significant interaction term (*P* = 0.8666) excluded effect modification, and stratified analyses only identified an elevated risk of a twin birth from blastocyst transfer in the DET subgroup, confirming that this discrepancy originated purely from confounding.

Multiple models have evaluated twin risk post-embryo transfer. Lan Y et al. reported a nomogram predicting twin and preterm birth risks following double cleavage-stage embryo transfer (16), yet their analysis enrolled unselected IVF patients, without focusing on advanced-age women or fully incorporating embryo-quality indices. Our nomogram specifically targets women aged ≥35 years and integrates embryo-related parameters to enable individualized pre-transfer estimation of twin live-birth risk for SET-versus-DET decision-making. Meta-analyses have demonstrated that DET increases multiple-gestation risk despite potential live-birth benefits in certain subgroups (17). Thus, clinicians must balance live-birth gains against twin-related morbidities even for older patients. Our model provides quantitative evidence for shared clinical decision-making and discourages empirical DET based purely on maternal age.

Prior studies have explored embryo transfer number among advanced age women. Alasmari et al. observed comparable live birth rates between SET and DET in women >40 years, whereas elective SET minimized multiple pregnancy risk (18, 19). Cai et al. reported similar cumulative live birth rates after single versus double blastocyst transfer in women >36 years (74.2% vs 63.0%; aOR 1.09, 95% CI 0.68–1.75), while single blastocyst transfer was associated with lower risks of preterm birth (aOR 0.37, 95% CI 0.21–0.64) and low birth weight (aOR 0.31, 95% CI 0.16–0.60) (20). McLernon et al. noted that two SET cycles achieved pregnancy rates comparable to one DET cycle, and high quality SET reduced multiple gestation risk (21). Moreover, singleton pregnancy after SET confers lower maternal healthcare costs and fewer maternal neonatal complications, preterm birth and low birth weight, relative to multiple gestation from DET (22). Given these perinatal risks and costs in older women (9), elective SET is generally recommended. Nevertheless, these reports did not account for embryo quality, a critical determinant of IVF outcomes.

The comparative outcomes of SET and DET stratified by embryo quality remain controversial. Aldemir et al. observed lower pregnancy and live-birth rates in mixed quality blastocyst transfers (good + poor quality) versus double good quality blastocyst transfers, with non-significant differences in multiple pregnancy rates; similarly, mixed-quality cleavage-stage transfers showed a non significant reduction in multiple gestation (23). However, participants in that study were predominantly <35 years old, and small event counts may have undermined result stability. Zeng et al. found comparable live-birth and multiple pregnancy rates between single good quality embryo transfer and DET (good + poor quality) among women >35 years (24). A meta analysis further demonstrated similar pregnancy and live birth rates between these two strategies, yet single good-quality embryo transfer yielded substantially lower risks of multiple pregnancy (RR = 0.14; 95% CI 0.09–0.20) and multiple birth (RR = 0.08; 95% CI 0.06–0.12) (25). In our cohort of women ≥35 years, embryo quality was also an independent risk factor for twin live birth, consistent with Lan et al (16). Discrepancies across published studies are likely attributable to inter study differences in maternal age and limited sample sizes. Collectively, these data support elective single good quality embryo transfer for women ≥35 years to mitigate twin risk. Evidence guiding transfer strategies for poor quality embryos remains scarce. Jiang et al. found no differences in pregnancy or multiple pregnancy rates between single and double poor quality blastocyst transfer in women ≥35 years, though double transfer significantly improved live-birth rates (11), which aligns with our observations. When both maternal age and embryo quality are considered, our prediction model may assist clinicians in appropriately adjusting embryo transfer number.

As is well known, age is an important factor affecting ART outcomes (26). Several studies have demonstrated a decline in oocyte quality in women older than 35 years (especially those older than 40 years) (26–28), leading to decreased embryo implantation and pregnancy rates (29). Lee et al. found that increasing age had a greater impact on pregnancy and multiple pregnancy rates than the morphological grade of the embryo in women older than 35 years (30). Jiao et al. reported that in individuals aged ≥36 years, the multiple pregnancy rate decreased significantly with increasing age (31). Our study also found that maternal age protected against a twin live birth. There are multiple reasons for the decline in ART outcomes with advancing age, including progressive depletion of oocytes, mitochondrial dysfunction, and an increased rate of embryo aneuploidy as a result of irregular meiosis (29, 32–34).

Controversy has long existed regarding the relationship between endometrial thickness and pregnancy outcomes. However, relatively few studies have focused on the effect of endometrial thickness on the multiple pregnancy rate. Lan et al. developed a prediction model for twin pregnancy after double cleavage-stage embryo transfer and demonstrated that endometrial thickness was a risk factor affecting the twin pregnancy rate (16). The findings of our present study also suggest that an endometrial thickness ≥7 mm is positively correlated with the risk of a twin live birth. In summary, endometrial thickness is one of the risk factors for multiple pregnancy; however, the threshold endometrial thickness for a twin live birth requires confirmation in further research.

The high OR for DET (OR = 19.45) warrants careful interpretation. In a prediction model, regression coefficients represent adjusted associations rather than causal effects. The large magnitude is largely expected from a biological perspective, because twin live birth generally requires the implantation of two transferred embryos; this consistency supports the face validity of the model. In addition, because the number of embryos transferred is selected according to patient prognosis, indication based confounding cannot be fully excluded, and residual confounding may influence the estimate. Importantly, the nomogram is intended to predict the probability of twin live birth under a selected transfer strategy, rather than to quantify the causal effect of switching from single to double embryo transfer, which would require causal inference methods beyond the scope of this study.

This research had some limitations. First, this study adopted a retrospective design, which inevitably introduced inherent biases. Nevertheless, multivariate logistic regression and multiple adjusted models strengthened the reliability of our results. Second, the present model was established only for women aged ≥35 years, and its applicability in younger populations requires further verification. Third, this was a single-center study with only bootstrap internal validation, lacking multicenter external or temporal validation. Although bootstrap internal validation can quantify overfitting within the original dataset, it tends to yield optimistic performance estimates and cannot fully confirm the model's generalizability to external cohorts or different clinical settings. Differences in embryo grading between the various guidelines and laboratories may lead to variability in embryo assessment. Considering that this was a single center study and that our laboratory follows strict standard operating procedures, our grading system was relatively stable overall, and differences among embryologists and grading guidelines had minimal impact on the results. We will perform a multicenter prospective independent cohort study in the future for external validation of our prediction model and for confirmation of its generalizability.

In summary, we found that the risk of a twin live birth correlated positively with two embryos transferred, two good quality embryos, blastocyst transfer, and endometrial thickness ≥7 mm and correlated negatively with maternal age. Fewer embryos transferred for younger patients may decrease the risk of a twin live birth. Based on our single center retrospective cohort, these predictors can identify patients at high risk of this outcome. Our internally validated nomogram allows reliable personalized pre-transfer risk stratification. However, external validation is needed for broader application.

## Data Availability

The raw data supporting the conclusions of this article will be made available by the authors, without undue reservation.
