# Supplementary material for: A model for prediction of a twin live birth following in vitro fertilization–embryo transfer in women of advanced age
Source: Front Med (Lausanne). 2026 Oct 13;13:1789377. doi: 10.3389/fmed.2026.1789377 (PMC13710278; doi:10.3389/fmed.2026.1789377)
Supplement: Supplementary file 1 [file Datasheet1.docx]

## Table S1 Multiple Regression Analysis Including Cases of Foetal Reduction

| Items | OR(95%CI) | P value |
| --- | --- | --- |
| Age | 0.70 (0.66, 0.74) | <0.001 |
| Basal FSH (mIU/ml) | 0.97 (0.92, 1.01) | 0.147 |
| Basal E2 (pg/ml) | 1.00 (0.99, 1.00) | 0.129 |
| AMH（ng/ml） | 1.04 (0.99, 1.09) | 0.105 |
| Number of oocytes retrieved | 0.99 (0.96, 1.02) | 0.503 |
| Endometrial thickness (mm) |  |  |
| <7 | 1 |  |
| >=7 | 2.41 (1.18, 4.95) | 0.016 |
| Number of good quality embryos transferred |  |  |
| ≤1 | 1 |  |
| 2 | 2.14 (1.67, 2.73) | <0.001 |
| Transferred embryo number |  |  |
| 1 | 1 |  |
| 2 | 18.05 (10.75, 30.30) | <0.001 |
| Embryo type |  |  |
| Cleavage-stage embryo | 1 |  |
| Blastocyst | 1.78 (1.30, 2.46) | <0.001 |
| Number of usable embryos |  |  |
| <=4 | 1 |  |
| >4, <=9 | 1.01 (0.76, 1.34) | 0.936 |
| >9, <=15 | 1.71 (0.97, 3.01) | 0.064 |
| >15 | 3.05 (0.79, 11.86) | 0.107 |
| Type of infertility |  |  |
| Secondary | 1 |  |
| Primary | 1.03 (0.80, 1.31) | 0.827 |

Fig. S1 ROC curve for assessing the accuracy of twin live birth Including Cases of Foetal Reduction

Note：Endometrial thickness:0 =＜7 mm,1 =≥7 mm；Number of good quality embryos:1 = 0/1 good quality embryos, 0 = 2 good quality embryo;Embryo type:1 = cleavage-stage embryo, 0 = blastocyst;transferred embryo number:0= 1embryos ;1=2 embryos

****Fig.** S2**  ROC curve for assessing the accuracy of twin live birth Including Cases of Foetal Reduction

4a

4b

****Fig.** S3 a** Calibration curve for the training sample (Calibration for training sample). The calibration curve was used to assess model calibration; X-axis: model-predicted twin live birth probability; Y-axis: observed twin live birth probability. The red diagonal represents ideal perfect calibration, with the black fitted curve enclosed by yellow 95% CI shading.****Fig.** S3 b** Decision curve analysis for the training cohort. Blue curve: net benefit of the predictive model; red curve: universal embryo transfer; green curve: no embryo transfer.

Note: The training-set calibration curve exhibited good consistency between predicted and observed twin live birth probabilities below 0.18. Risk was slightly overestimated for probabilities >0.18 with wider confidence intervals, owing to limited high-risk cycles.

Decision curve analysis demonstrated the model achieved greater net benefit than universal/no transfer strategies at thresholds of 0–20%.

Table S2 Stratified prediction results for twin live birth according to SET and DET (cleavage-stage embryos versus blastocysts)

| ID | Age (years) | Endometrial thickness (mm)(0=<7mm；1=>=7mm) | Number of good quality embryos (1=0/1，0 =2) | Cleavage-stage embryo | | Blastocyst | |
| --- | --- | --- | --- | --- | --- | --- | --- |
|  |  |  |  | Predicted twin live birth risk under SET(p) | Predicted twin live birth risk under DET(p) | Predicted twin live birth risk under SET(p) | Predicted twin live birth risk under DET(p) |
| 1 | 35 | 1 | 1 | 0.55% | 9.56% | 1.17% | 18.52% |
| 2 | 36 | 1 | 1 | 0.38% | 6.76% | 0.80% | 13.48% |
| 3 | 38 | 0 | 0 | 0.14% | 2.55% | 0.29% | 5.32% |
| 4 | 39 | 1 | 0 | 0.28% | 5.04% | 0.59% | 10.24% |
| 5 | 40 | 1 | 1 | 0.08% | 1.58% | 0.18% | 3.33% |
| 6 | 43 | 0 | 0 | 0.02% | 0.39% | 0.04% | 0.84% |

## Note:Probabilities of twin live birth were calculated using the established prediction model: logit(p)=ln(p/1-p) =8.51323 -0.37756age +2.95569(transferred embryo number=2) -0.76533*(Cleavage-stage embryo)+1.08475*(Endometrial thickness ≥ 7mm)-0.82053*(number of good quality embryos= 0/1), In the formula, p denotes the predicted probability of twin live birth.

## Table S3 Multiple Regression Analysis Including Interaction term

| Items | OR(95%CI) |  | *P* value |
| --- | --- | --- | --- |
| Age | 0.69 (0.66, 0.73) |  | <0.001 |
| Basal FSH (mIU/ml) | 0.97 (0.92, 1.01) |  | 0.127 |
| Basal E2 (pg/ml) | 0.99 (0.99, 1.00) |  | 0.105 |
| AMH（ng/ml） | 1.04 (0.99, 1.09) |  | 0.119 |
| Number of oocytes retrieved | 0.99 (0.96, 1.02) |  | 0.633 |
| Endometrial thickness (mm) |  |  |  |
| <7 | 1 |  |  |
| >=7 | 2.79 (1.30, 5.99) |  | 0.009 |
| Number of good quality embryos transferred |  |  |  |
| ≤1 | 1 |  |  |
| 2 | 2.20 (1.72, 2.83) |  | <0.001 |
| Transferred embryo number |  |  |  |
| 1 | 1 |  |  |
| 2 | 18.05 (6.61, 49.30) |  | <0.001 |
| Embryo type |  |  |  |
| Cleavage-stage embryo | 1 |  |  |
| Blastocyst | 1.72 (0.55, 5.33) |  | 0.351 |
| Number of usable embryos |  |  |  |
| <=4 | 1 |  |  |
| >4, <=9 | 1.00 (0.75, 1.34) |  | 0.992 |
| >9, <=15 | 1.74 (0.98, 3.07) |  | 0.059 |
| >15 | 2.78 (0.71, 10.86) |  | 0.142 |
| Type of infertility |  |  |  |
| Secondary | 1 |  |  |
| Primary | 1.03 (0.80, 1.32) |  | 0.832 |
| Interaction term |  |  |  |
| 0 | 1 |  |  |
| 1 | 1.10 (0.35, 3.53) |  | 0.867 |

Note: Interaction term (transferred embryo number × embryo type): 1 = transfer of two blastocysts; 0 = transfer of one cleavage embryo, two cleavage embryos, or one blastocyst.

Table S4 Univariate Analyses of Twin Live Birth-Related Factors According to the Number of Transferred Embryos

| Items | One embryos transferred | |  | | Two embryos transferred | |  |
| --- | --- | --- | --- | --- | --- | --- | --- |
|  | OR(95%CI) | *P* value |  |  | OR(95%CI) | *P* value |  |
| Age | 0.9 (0.7, 1.1) | 0.337 | | 0.7 (0.6, 0.7) | | <0.001 |  |
| BMI | 0.9 (0.8, 1.0) | 0.214 | | 1.0 (0.9, 1.0) | | 0.242 |  |
| Duration of infertility | 1.0 (0.9, 1.1) | 0.495 | | 1.0 (1.0, 1.0) | | 0.376 |  |
| Basal FSH (mIU/ml) | 1.0 (0.8, 1.2) | 0.769 | | 0.9 (0.9, 0.9) | | <0.001 |  |
| Basal LH (mIU/ml) | 1.1 (1.0, 1.2) | 0.101 | | 1.0 (1.0, 1.1) | | 0.202 |  |
| Basal E2 (pg/ml) | 1.0 (1.0, 1.0) | 0.339 | | 1.0 (1.0, 1.0) | | 0.027 |  |
| AMH（ng/ml） | 1.1 (1.0, 1.2) | 0.220 | | 1.2 (1.1, 1.2) | | <0.001 |  |
| Number of oocytes retrieved | 1.1 (1.0, 1.2) | 0.010 | | 1.1 (1.1, 1.1) | | <0.001 |  |
| Endometrial thickness(mm) |  |  | |  | |  |  |
| <7 | 1 |  | | 1 | |  |  |
| >=7 | 1.0 (0.1, 7.6) | 0.993 | | 4.0 (1.7, 9.0) | | <0.001 |  |
| Type of infertility |  |  | |  | |  |  |
| Secondary infertility | 1 |  | | 1 | |  |  |
| Primary infertility | 3.4 (1.3, 8.9) | 0.013 | | 1.5 (1.2, 2.0) | | 0.001 |  |
| Number of good quality embryos transferred |  |  | |  | |  |  |
| 0 | 1 |  | | 1 | |  |  |
| 1 | 1.7 (0.6, 4.8) | 0.317 | | 1.0 (0.7, 1.4) | | 0.847 |  |
| 2 | NA |  | | 1.7 (1.2, 2.3) | |  | |
| transferred embryo number |  |  | |  | |  | |
| 1 |  |  | |  | |  | |
| 2 |  |  | |  | |  | |
| Embryo type |  |  | |  | |  | |
| Cleavage-stage embryo | 1 |  | | 1 | |  | |
| Blastocyst | 2.4 (0.8, 7.2) | 0.134 | | 1.8 (1.4, 2.3) | | <0.001 | |
| Number of usable embryos |  |  | |  | |  | |
| <=4 | 1 |  | | 1 | |  | |
| >4, <=9 | 1.6 (0.5, 4.9) | 0.419 | | 1.7 (1.3, 2.1) | | <0.001 | |
| >9, <=15 | 3.0 (0.6, 14.3) | 0.166 | | 3.4 (2.2, 5.4) | | <0.001 | |
| >15 | 20.7 (4.3, 99.6) | <0.001 | | 1.6 (0.2, 13.1) | | 0.643 | |

Table S5 Multivariate Binary Logistic Regression for Predicting Twin Live Birth Stratified by SET and DET

| Exposure | SET | |  | | DET | |
| --- | --- | --- | --- | --- | --- | --- |
|  | OR(95%CI) | *P* value |  |  | OR(95%CI) | *P* value |
| Age (years) | 0.92 (0.76, 1.11) | 0.385 | | 0.67 (0.64, 0.71) | | <0.001 |
| Endometrial thickness (mm)l |  |  | |  | |  |
| <7 | 1.0 |  | | 1.0 | |  |
| >=7 | 0.99 (0.13, 7.49) | 0.991 | | 3.30 (1.45, 7.53) | | 0.005 |
| Number of good quality embryos |  |  | |  | | <0.001 |
| ≤1 | - | - | | 1.0 | |  |
| 2 | - | - | | 2.28 (1.78, 2.92) | |  |
| Embryo type |  |  | |  | | <0.001 |
| Cleavage-stage embryo | 1.0 |  | | 1.0 | |  |
| Blastocyst | 2.20 (0.71, 6.82) | 0.171 | | 2.15 (1.60, 2.89) | |  |

Table S6 Dummy coding of categorical variables

| Variable | Coding definition |
| --- | --- |
| Endometrial thickness | 0 = ＜7 mm; 1 = ≥7 mm |
| Number of good quality embryos | ≤1 = 0/1 good quality embryos; 0 = 2 good quality embryo |
| Embryo type | 1 = cleavage-stage embryo; 0 = blastocyst; |
| Number of embryos transferred | 0= 1embryos ; 1=2 embryos |

Table S7 Factors associated with twin live birth: univariate and multivariate GEE analyses

| Variable | Univariate analysis | | Multivariate analysis | |
| --- | --- | --- | --- | --- |
|  | OR(95%CI) | P-value | OR(95%CI) | P-value |
| Age | 0.71 (0.68, 0.75) | <0.001 | 0.70 (0.66, 0.74) | <0.001 |
| Type of infertility |  |  |  |  |
| Secondary infertility | 1 |  | 1 |  |
| Primary infertility | 1.62 (1.28, 2.06) | <0.001 | 1.03 (0.80, 1.33) | 0.813 |
| Basal FSH (mIU/ml) | 0.91 (0.88, 0.95) | <0.001 | 0.96 (0.92, 1.01) | 0.085 |
| Basal E2 (pg/ml) | 0.99 (0.99, 1.00) | 0.005 | 0.99 (0.99, 1.00) | 0.089 |
| AMH（ng/ml） | 1.12 (1.09, 1.16) | <0.001 | 1.04 (0.99, 1.09) | 0.097 |
| Number of oocytes retrieved | 1.05 (1.04, 1.07) | <0.001 | 0.99 (0.96, 1.02) | 0.684 |
| Number of usable embryos |  |  |  |  |
| <=4 | 1 |  | 1 |  |
| >4, <=9 | 1.39 (1.10, 1.75) | 0.006 | 0.99 (0.73, 1.34) | 0.950 |
| >9, <=15 | 2.14 (1.43, 3.22) | <0.001 | 1.70 (0.95, 3.05) | 0.076 |
| >15 | 1.94 (0.61, 6.17) | 0.260 | 2.70 (0.65, 11.24) | 0.172 |
| Endometrial thickness(mm) |  |  |  |  |
| <7 | 1 |  | 1 |  |
| >=7 | 3.47 (1.63, 7.39) | 0.001 | 2.77 (1.26, 6.08) | 0.011 |
| Number of good quality embryos transferred |  |  |  |  |
| 1 | 1 |  | 1 |  |
| 2 | 3.70 (3.00, 4.56) | <0.001 | 2.19 (1.69, 2.83) | <0.001 |
| transferred embryo number |  |  |  |  |
| 1 | 1 |  | 1 |  |
| 2 | 15.79 (9.69, 25.72) | <0.001 | 18.93 (11.27,31.81) | <0.001 |
| Embryo type |  |  |  |  |
| Cleavage-stage embryo | 1 |  | 1 |  |
| Blastocyst | 0.67 (0.53, 0.85) | 0.001 | 1.84 (1.31, 2.57) | <0.001 |

Note: Generalized estimating equations (GEE) were performed to account for clustering caused by repeated embryo‑transfer cycles from the same patient

Table S8 Multivariable regression model after multiple imputation

| Variable | 95%CI | P-value |
| --- | --- | --- |
| Age | 0.70 (0.70, 0.70) | <0.001 |
| Type of infertility |  |  |
| Secondary infertility | 1 |  |
| Primary infertility | 1.00 (0.78, 1.28) | 1.000 |
| Basal FSH (mIU/ml) | 1.00 (0.95, 1.05) | 1.000 |
| Basal E2 (pg/ml) | 1.00 (1.00, 1.00) | 1.000 |
| AMH（ng/ml） | 1.00 (0.95, 1.05) | 1.000 |
| Number of oocytes retrieved | 1.00 (1.00, 1.00) | 1.000 |
| Number of usable embryos |  |  |
| <=4 | 1 |  |
| >4, <=9 | 1.00 (0.78, 1.29) | 1.000 |
| >9, <=15 | 1.70 (0.97, 2.97) | 0.062 |
| >15 | 2.98 (0.78, 11.40) | 0.111 |
| Endometrial thickness(mm) |  |  |
| <7 | 1 |  |
| >=7 | 2.40 (1.18, 4.89) | 0.016 |
| Number of good quality embryos transferred |  |  |
| 1 | 1 |  |
| 2 | 2.10 (1.67, 2.65) | <0.001 |
| transferred embryo number |  |  |
| 1 | 1 |  |
| 2 | 17.796 (10.59, 29.90) | <0.001 |
| Embryo type |  |  |
| Cleavage-stage embryo | 1 |  |
| Blastocyst | 1.80 (1.31, 2.47) | <0.001 |
